# Childhood attention deficit hyperactivity disorder traits, societal exclusion and midlife psychological distress

**DOI:** 10.1038/s44220-026-00600-0

**Published:** 2026-03-27

**Authors:** Amber John, Elizabeth O’Nions, Lucy Corrigan, Joanne Cotton, Warren James Donnellan, Danielle Nimmons, Henry Shelford, Aphrodite Eshetu, Rob Saunders, Celine El Baou, Gavin R. Stewart, Rachael W. Cheung, Roopal Desai, Douglas GJ. McKechnie, Jae Won Suh, William Mandy, Darya Gaysina, Georgia Pavlopoulou, Philip Asherson, Jessica Agnew-Blais, Joshua Stott

**Affiliations:** 1 Department of Psychology, University of Liverpool, Liverpool, UK; 2 Clinical, Educational, and Health Psychology (CEHP), University College London, London, UK; 3 Bradford Institute for Health Research, Bradford Teaching Hospitals NHS Foundation Trust, Bradford, UK; 4 Department of Primary Care and Population Health, University College London, London, UK; 5 ADHD UK, London, UK; 6 Social, Genetic and Developmental Psychiatry Centre, Kings College London, London, UK; 7 School of Psychology, University of Sussex, Sussex, UK; 8 Group for Research in Relationships and NeuroDiversity (GRRAND), Brain Sciences, University College London, London, UK; 9 Anna Freud Center, London, UK; 10 Department of Psychology, Queen Mary University of London, London, UK

**Keywords:** Psychology, ADHD

## Abstract

Most research on attention deficit hyperactivity disorder (ADHD) focuses on childhood or early adulthood. Less is known about the impact of childhood ADHD traits across the lifespan. This study aims to test (1) whether childhood ADHD traits are associated with trajectories of psychological distress across adulthood up to midlife, and (2) the role of societal exclusion in the relationship between ADHD traits and midlife distress. Data were from the 1970 British Cohort Study (*N* = 17,196 at birth), a prospective longitudinal cohort. ADHD traits were measured at age 10 using a validated 14-item measure. Psychological distress was assessed at 5 time points (26–46) using the Malaise Inventory Scale. Measures of 5 domains of societal exclusion (health, relational, political, economic and services) were available at age 34. Higher childhood ADHD traits were associated with higher distress across adulthood and being in higher distress trajectories. The predicted probability of having clinically relevant distress in midlife was about 27% for people who had high childhood ADHD traits (5.05%), compared with 18% for those who did not have high ADHD traits, adjusting for sex, ethnicity and childhood social class. Societal exclusion acted as an indirect pathway in the association between ADHD traits and midlife psychological distress through health, relational, economic and services exclusion, but not political exclusion. People with higher childhood ADHD traits are more likely to experience psychological distress in adulthood, which was partly explained by societal exclusion. Exclusion experienced by people with ADHD may be a determinant of long-term adverse mental health outcomes. Addressing structural and relational barriers across the life course is an important step toward promoting well-being for people with ADHD.

## Abstract (web summary)

In this population-based cohort study, John et al. evaluated the long-term impact of childhood ADHD traits on adult psychological distress and identified societal exclusion as an indirect pathway linking early ADHD traits to poorer midlife mental health.

## Main

Attention deficit hyperactivity disorder (ADHD) is a form of neurodivergence characterized by differences in attention, activity and impulse control. As a result, people with ADHD can experience both challenges in daily functioning^1^ and key strengths^2^, although these experiences vary across individuals. Global prevalence of ADHD is around 8.0% in children–adolescents^3^ and 3.1% in adults^4^. ADHD traits frequently persist into adulthood^5^ and are associated with a range of negative life outcomes, including lower educational attainment^6^, employment difficulties^7^, poorer physical health^8^ and earlier mortality^9^.

In particular, people with ADHD are more likely to experience mental health problems and psychological distress^10^. However, most research has focused on childhood and early adulthood, meaning less known about how early ADHD traits shape long-term patterns of distress in adulthood up to middle age. In addition, research has not yet focused on understanding heterogeneity in distress outcomes across adulthood in people with ADHD. For example, some people with ADHD may experience persistent distress over the life course, whereas others may experience fluctuating patterns of distress over time. Understanding these trajectories is important to identify groups of people at particular risk of experiencing distress and informing early intervention strategies for those with ADHD.

The mechanisms underlying potential associations between ADHD traits and distress are unclear, but may relate, in part, to adverse life experiences of people with ADHD across the lifespan. Societal exclusion is a complex construct that can be defined in different ways. In this study, we define societal exclusion as the experience of systemic disadvantage occurring across multiple domains, preventing people from participating fully in life and society^11^. Domains of societal exclusion include employment, social relationships, political engagement, access to resources and public services^12^. Evidence suggests that people with ADHD traits are more likely to experience exclusion across these domains^13,14^. There is further research that has shown that experiences of societal exclusion may, in turn, be associated with distress^15^. However, to our knowledge, the hypothesis that societal exclusion may partially explain associations between ADHD traits and distress has not yet been formally tested using longitudinal data. Understanding societal exclusion in this context is important, because it may represent a key target for interventions aimed at improving longer-term mental health outcomes in people with ADHD.

This study aims to test (1) whether childhood ADHD traits are associated with cumulative and trajectory-based patterns of psychological distress across adulthood, and (2) whether domains of societal exclusion in adulthood act as pathways underlying any association between ADHD traits and distress in midlife. This can offer new insight into long-term mental health implications of early ADHD traits and whether societal exclusion acts as a potentially modifiable mechanism of this association.

## Results

### Descriptive statistics and missing data

A total of 9,280 people were included in the main path model. Missing data analysis comparing this sample with the sample excluded from the main model owing to missing data on at least one key variable or covariate showed key differences between the groups (Supplementary Table 1). In brief, compared with the sample included in the main models, the sample with missing data was more likely to have higher ADHD traits and higher levels of health, relational and economic exclusion. They were also more likely to be men, minority ethnic groups and have lower socioeconomic position. Descriptive statistics for the main analytic sample are presented in Table 1.

**Table 1:** Descriptive statistics for analytic sample

	Descriptives	Range
ADHD traits*, mean (s.d.)	−0.12 (0.85)	−1.12–2.57
Psychological distress at age 46, mean (s.d.)	1.75 (2.11)	0–9
Health exclusion, mean (s.d.)	0.56 (0.90)	0–4
Relational exclusion, mean (s.d.)	0.53 (0.67)	0–3
Political exclusion, mean (s.d.)	2.17 (1.15)	0–4
Economic exclusion, mean (s.d.)	1.06 (1.22)	0–5
Service exclusion, mean (s.d.)	0.71 (1.01)	0–5
Sex, *N* (%)		
Male	4,502 (48.51)	.
Female	4,778 (51.49)	.
Ethnicity, *N* (%)		
White	9,007 (97.06)	.
Minoritized ethnicity	273 (2.94)	.
Social class at age 10, *N* (%)		
Unskilled	350 (3.77)	.
Partly skilled	1,202 (12.95)	.
Manual	3,752 (40.43)	.
Non-manual	1,040 (11.21)	.
Managerial and technical	2,343 (25.25)	.
Professional	593 (6.39)	.

^*^Dimensional score of ADHD traits.

### Trajectories of distress

**Fig. 1:**
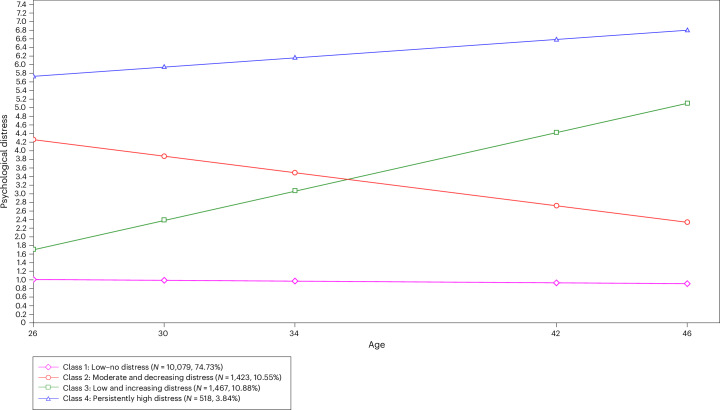
Trajectories of psychological distress from ages 26 to 46 estimated growth mixture models. Estimated trajectories of psychological distress from ages 26 to 46 derived from growth mixture modeling. Four classes were identified: low–no distress, moderate and decreasing distress, low and increasing distress and persistently high distress.

Growth curve models showed that fit statistics were similar for linear and quadratic models (linear, comparative fit index (CFI) = 0.97, Tucker–Lewis index (TLI) = 0.97, root mean square error of approximation (RMSEA) = 0.06, standardized root mean square residual (SRMR) = 0.03; quadratic, CFI = 0.98, TLI = 0.96, RMSEA = 0.06, SRMR = 0.03). As such, the most parsimonious model (linear) was selected for further analyses. Growth mixture models were then fitted to the data to compare the 2-, 3-, 4-, 5- and 6-class models. Results showed that the 4-class model provided the optimal solution, in line with previous research deriving similar trajectories in these data^16^. Detailed information about class comparisons is presented in Supplementary Material 2 and Supplementary Table 2. Trajectories of distress from ages 26 to 46 estimated from the 4-class model are presented in Fig. 1. Trajectories identified are (1) low–no distress, (2) moderate and decreasing distress, (3) low and increasing distress, and (4) persistently high distress.

### Associations between ADHD traits at age 10 and distress from ages 26 to 46

Linear regression models showed that ADHD traits at age 10 were significantly associated with a greater proportion of time points with clinically relevant distress (unadjusted, regression coefficient (*B*) = 0.11, 95% confidence intervals (CI) = 0.09–0.13), *P* < 0.001; adjusted, *B* = 0.13, 95% CI = 0.10–0.15, *P* < 0.001) (Table 2). Next, multinomial logistic regressions were run to test associations between ADHD traits and distress class membership. Results showed that compared with being in the no–low distress group, people with high ADHD traits were significantly more likely to fall into higher distress groups, including the moderate and decreasing (unadjusted, relative risk ratio (RRR) = 1.25, 95% CI = 1.17–1.34, *P* < 0.001; adjusted, RRR = 1.38, 95% CI = 1.28–1.48, *P* < 0.001), low and increasing (unadjusted, RRR = 1.23, 95% CI = 1.15–1.31, *P* < 0.001; adjusted, RRR = 1.30, 95% CI = 1.21–1.40, *P* < 0.001), and the persistently high (unadjusted, RRR = 1.46, 95% CI = 1.32–1.62, *P* < 0.001; adjusted, RRR = 1.46, 95% CI = 1.31–1.64, *P* < 0.001) distress groups (Table 3).

**Table 2:** Associations between ADHD traits at age 10 and proportion of time points with clinically relevant psychological distress up to age 46 (linear regression)

	Model 1: unadjusted	Model 2: fully adjusted
ADHD traits at age 10	0.11 (0.09–0.13), <0.001*	0.13 (0.10–0.15), <0.001

Results for covariates are shown in Supplementary Table 3, instead of main paper, to avoid ‘Table 2 fallacy’^24^.

**Table 3:** Associations between ADHD traits at age 10 and psychological distress class membership (multinomial logistic regressions)

	Model 1: unadjusted	Model 2: fully adjusted
Class 2: moderate and decreasing distress*		
ADHD traits at age 10	1.25 (1.17–1.34), <0.001**	1.38 (1.28–1.48), <0.001
**Class 3: low and increasing distress**		
ADHD traits at age 10	1.23 (1.15–1.31), <0.001	1.30 (1.21–1.40), <0.001
**Class 4: persistently high distress**		
ADHD traits at age 10	1.46 (1.32–1.62), <0.001	1.46 (1.31–1.64), <0.001

*Reference category: low–no distress (class 1).

**Presented as RRR (95% CI), *P*.

Results for covariates are shown in Supplementary Table 3, instead of main paper, to avoid ‘Table 2 fallacy’^24^.

Margins analysis from a logistic regression model using the binary indicator of ADHD traits showed that individuals with high likelihood of meeting Diagnostic and Statistical Manual of Mental Disorders, 5th edition (DSM-5) (ref. ^1^) criteria for ADHD (comprising 5.05% of the sample) had a predicted probability of 26.74% (95% CI = 21.72–31.76%) of having clinically relevant distress at age 46, compared with 18.32% (95% CI = 17.37–19.27%) for those without high ADHD traits. The absolute risk difference was 8.42 percentage points.

### The role of societal exclusion in the association between ADHD traits and midlife distress

**Fig. 2:**
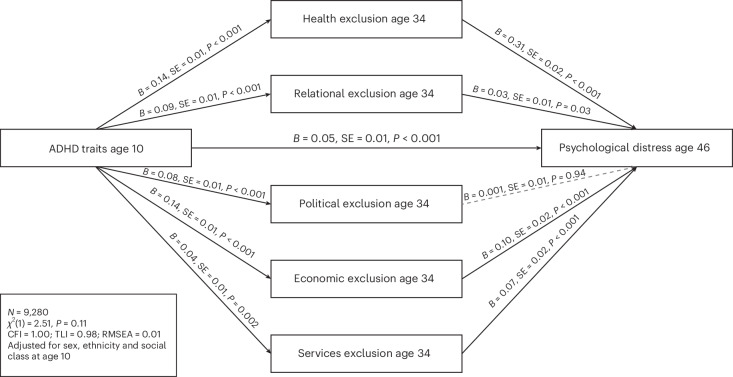
Path model testing the role of societal exclusion in the association between ADHD traits and midlife psychological distress. Path model testing associations between ADHD traits at age 10, domains of societal exclusion at age 34 (health, relational, political, economic and services exclusion) and psychological distress at age 46. Regression coefficients, standard errors and *P* values are shown. The model was adjusted for sex, ethnicity and social class at age 10 (*N* = 9,280). Solid lines indicate statistically significant paths. Dashed lines indicate non-significant paths.

The adjusted path model (*N* = 9,280) fit the data well (chi-square (*χ*^2^ ) = 2.51, degrees of freedom (df) = 1, *P* = 0.11; CFI = 1.00; TLI = 0.98; RMSEA = 0.01). Results showed a significant total effect for associations between ADHD and distress at age 46 (*B* = 0.11, standard error (SE) = 0.01, *P* < 0.001). There was a significant direct effect of ADHD traits on distress at age 46 (*B* = 0.05, SE = 0.01, *P* < 0.001). There were also significant indirect effects of ADHD on distress through societal exclusion in all domains (health exclusion, *B* = 0.04, SE = 0.004, *P* < 0.001; relational exclusion, *B* = 0.003, SE = 0.001, *P* = 0.04; economic exclusion, *B* = 0.01, SE = 0.003, *P* < 0.001; services exclusion, *B* = 0.003, SE = 0.001, *P* = 0.01), except for political exclusion (*B* = 0.00, SE = 0.001, *P* = 0.94). ADHD traits were significantly associated with higher levels of political exclusion (*B* = 0.08, SE = 0.01, *P* < 0.001), but this was not, in turn, associated with levels of distress at age 46 (*B* = 0.001, SE = 0.01, *P* = 0.94). Results also showed a significant total indirect effect of associations between ADHD traits and midlife distress through societal exclusion (*B* = 0.06, SE = 0.01, *P* < 0.001) (Fig. 2). The unadjusted model was consistent (Supplementary Fig. 1).

## Discussion

### Summary and interpretation of findings

This study tests the patterns of distress in adulthood in people with childhood ADHD traits and the role of societal exclusion in this relationship. Using data from the 1970 British Cohort Study, we found that higher ADHD traits at age 10 were significantly associated with an increased proportion of time points with clinically relevant psychological distress between ages 26 and 46. Growth mixture modeling identified four distinct trajectories of distress from ages 26 to 46: (1) low–no distress, (2) moderate and decreasing distress, (3) low and increasing distress, and (4) persistently high distress. People with higher ADHD traits were more likely to belong to the moderate and decreasing, low and increasing, and persistently high distress trajectories, compared with the low–no distress group. The largest effect sizes were observed for the high distress group. Post-estimation showed that people with high childhood ADHD traits had a predicted probability of 27% of having clinically relevant distress at age 46, compared with 18% of people who did not have high ADHD traits. Taken together, these findings show that ADHD traits are associated with cumulative life course patterns of distress persisting through to midlife, although effect sizes are modest. One key finding is that ADHD traits were not only associated with worsening or persistent distress trajectories but also with the trajectory indicating moderate and decreasing patterns of distress. This may reflect patterns of increasing resilience with time, potentially owing to supportive environments or effective coping strategies and/or interventions. Further qualitative research could improve understanding of this, particularly key protective factors that may predict resilience and better long-term psychological outcomes in people with ADHD.

In addition, results showed that the association between childhood ADHD traits and distress at age 46 partially operated through societal exclusion across multiple domains measured at age 34, including health, relational, economic and service exclusions. These findings are consistent with emerging theories of cumulative disadvantage, where early vulnerabilities interact with social structures and life events to compound risk over time^17^. Higher ADHD traits were associated with increased political exclusion; however, political exclusion was not associated, in turn, with midlife distress. This suggests that more immediate or tangible forms of exclusion (such as access to care or secure employment) may play a greater role in shaping mental health outcomes.

Altogether, these findings support previous cross-sectional and longitudinal research showing associations between ADHD and poorer mental health outcomes (including emotional problems, depression and anxiety)^8,10,18,19^. These results further extend this by showing that childhood ADHD traits are associated with cumulative, longitudinal patterns of distress up to midlife, and that these associations are explained in part by domains of societal exclusion. Different domains of societal exclusion may reflect distinct mechanisms through which ADHD traits may be associated with adult distress. Health exclusion may reflect cumulative unmet health needs and limited access to healthcare, leading to poorer mental health and greater distress. Relational exclusion may increase distress by limiting access to protective social relationships and networks, as well as reducing overall sense of belonging. Economic exclusion (such as unemployment or job insecurity) may contribute to distress outcomes by increasing stress levels over time and reducing stability. Services exclusion may limit access to high-quality local services (for example, policing, education, public transport and so on), exacerbating existing inequalities and further increasing distress. Together, these findings suggest that the adverse mental health outcomes associated with childhood ADHD traits may be partly attributed to experiences of societal exclusion across multiple domains. These findings suggest that some of the key challenges faced by neurodivergent people, including people with childhood ADHD traits, arise as a result of exclusionary social structures and systemic barriers.

### Strengths and limitations

This study used data from the 1970 British Cohort Study, a cohort comprising a nationally representative sample of people born during that period, with follow-up spanning from birth to midlife. Repeated measures of psychological distress were available over this time, meaning that long-term patterns of distress could be modeled. In addition, the study used a measure of ADHD traits, rather than diagnosis data. This means that a greater proportion of individuals at increased likelihood of meeting DSM-5 criteria for ADHD traits could be studied than if the sample were restricted to a diagnosed cohort.

However, ADHD traits were captured at a single time point only, meaning that persistence or changes in ADHD symptoms over time could not be considered. Societal exclusion was also measured at a single time point, meaning that dynamic and potentially bidirectional relationships between exclusion and distress could not be explored.

Data on ADHD diagnosis and medication use were not available in these data, which is a limitation given that treatment may influence later mental health trajectories. It is likely that very few cohort members received a diagnosis or pharmacological treatment for ADHD in childhood, given limited clinical recognition at the time^20^. It would be valuable for future research to focus on understanding whether obtaining a diagnosis of ADHD or receiving medication for this modifies the associations between ADHD traits and distress outcomes across adulthood. Although the ADHD traits measure has shown good psychometric properties, it does not necessarily correspond to a clinical diagnosis of ADHD and may not match all criteria used in modern diagnostic frameworks. An additional limitation is that childhood social class was based on the father’s occupation, in line with data collection conventions at the time. However, this may not fully capture socioeconomic position, particularly where maternal occupation or other socioeconomic indicators were more important.

This study reflects the experiences of a cohort born in 1970, who were raised during a period of low public and clinical awareness of ADHD. The context in which ADHD symptoms were experienced differs considerably from that of more recent cohorts. This may potentially limit the generalizability of findings to younger generations. However, these findings highlight how people with high childhood ADHD traits who are now in middle age may have experienced societal exclusion throughout their earlier years, with potential longer-term impacts on distress levels.

An additional limitation is the lack of ethnic diversity in the sample. Minoritized ethnic groups comprised less than 3% of the overall sample, limiting generalizability of findings to broader populations. It is likely that people with minoritized ethnicities face additional life adversity and exclusion, which may, in turn, lead to greater levels of distress. In addition to this, all minoritized ethnic groups were collapsed into a single category, owing to the small sample sizes available, meaning that heterogeneous experiences of different ethnic groups could not be considered. Future research should aim to replicate these findings in ethnically diverse samples.

As with all long-running longitudinal cohorts, there was attrition over time in this data. This may have introduced bias, whereby people with higher ADHD traits or higher levels of societal exclusion were more likely to drop out of the study, leading to a potential underestimation of associations. Although principled missing data methods (for example, full information maximum likelihood) were used in analyses to mitigate this, they cannot fully eliminate this possibility. Future research should aim to improve the retention and inclusion of under-represented groups in cohort studies, for example, by reducing study burden and actively involving participants in study design and delivery.

Finally, while a range of key variables were accounted for in analyses, it is likely that there are other factors that play an important role in this association, which were not included in this study. For example, experiences of trauma or bullying, parent–child relationships and school support systems may play a key role in the relationship between ADHD traits and poor mental health, either directly or indirectly via other mechanisms, such as societal exclusion. Childhood mental health problems were also not included in models, as these may play a mediating role in the association between childhood ADHD traits and later psychological distress, and as such may constitute overadjustment.

### Directions for future research

Future research should extend these findings by testing risk and protective factors for mental health outcomes across the life course in people with ADHD. There is high co-occurrence of childhood ADHD traits with other mental health problems in childhood, such as depression and anxiety. As such, future research could aim to explore the role of childhood mental health problems in this association. Qualitative research can help to provide a richer picture of lived experiences of distress across the life course in people with ADHD, and can help improve understanding of the key underlying mechanisms of this. For example, access to early diagnosis, educational support, implementation of reasonable adjustments, relational support to boost sense of belonging and social relationships, and self-regulation strategies may be protective. Identifying these factors may help to inform development of system-level interventions (for example, inclusive educational policies) to improve mental health in people with ADHD. Future research should examine how social policy and service provision prevents or exacerbates exclusion among people with ADHD. Particular attention is needed to understand how these exclusions are intensified at the intersection of gender, ethnicity and other identities, where individuals may face compounded stigma and barriers to support. This could support not only more inclusive and responsive systems of care but also a societal shift toward valuing neurodiversity and reducing stigma.

### Potential implications

Findings from this study highlight the importance of societal exclusion in various domains, such as healthcare access, economic stability and social relationships, as a contributor to distress among people with high ADHD traits. Policymakers should consider strategies comprising both individual- and system-level interventions to reduce exclusion of people with ADHD to improve their mental health outcomes. Crucially, fostering a culture of understanding and acceptance, rather than correction, toward adults with ADHD is essential. Policies that ensure equitable access to education, healthcare and meaningful social and economic participation could reduce mental health disparities experienced by people with ADHD. These policies require a cross-discipline approach and might include implementing additional support for people with ADHD at the school level for educational and social inclusion^21^, improved training and resources for healthcare professionals and school staff around ADHD for health inclusion^22^, and support from occupational therapists for work-related economic inclusion^23^.

## Conclusion

Results from this study show that childhood ADHD traits are prospectively associated with cumulative patterns of distress across adulthood through to midlife. These results also suggest that experiences of societal exclusion in adulthood act as pathways linking ADHD traits to long-term mental health outcomes. This highlights the need for early neurodevelopmental support and sustained efforts to foster inclusive environments across the life course for people with ADHD. Addressing structural and relational barriers that people with ADHD traits face across the life course is an important step toward promoting their psychological well-being.

## Methods

This study complies with all relevant ethical regulations. All participants provided written informed consent. Ethical approval for the most recent wave of data collection for this data was obtained from the South East Coast—Brighton and Sussex MREC (15/LO/1446). Information about ethical approvals obtained for previous waves of data collection can be accessed online: https://cls.ucl.ac.uk/wp-content/uploads/2017/07/BCS70-Ethical-review-and-Consent-2019.pdf.

### Data

This study used data from the 1970 British Cohort Study (BCS70), a nationally representative longitudinal cohort study of 17,198 individuals born in England, Scotland and Wales during a single week in 1970 (ref. ^25^). Owing to attrition and missing data over follow-up, the number of participants with valid data for analyses was lower than the original 17,198 participants enrolled at birth. The main analytic sample comprised 9,280 participants. Characteristics of the analytic sample and differences to the sample with missing data are described in detail in ‘Results’. Follow-up data have been collected over 46 years, capturing a broad range of demographic, health and lifestyle information. Further details on data collection methods and participation rates at each wave have been published previously.

### Measures

#### ADHD traits

ADHD traits were derived using a validated 14-item measure based on items collected at age 10 (ref. ^26^) (9 items corresponding to hyperactivity and 5 to inattention). These items were part of standard behavior questionnaires completed by parents and teachers in BCS70 (refs. ^27,28^). Cotton and Baker (2019) applied a data mining framework to map these items from BCS70 onto the DSM-5 criteria for ADHD, identifying 14 items that corresponded closely to these criteria. A zero-inflated item response theory mixture model was used to derive a continuous dimensional score of ADHD traits, alongside a binary indicator of high ADHD traits based on DSM-5-aligned thresholds (*N* = 469, 5.05% of analytic sample met criteria). This approach demonstrated strong model fit and discriminative validity, with the derived scale showing expected associations with known correlates of ADHD, including male sex, social disadvantage, and later educational and behavioral outcomes^26^. Psychometric evaluation has shown strong reliability and validity of this measure, with a Kaiser–Meyer–Olkin value of 0.90 and Cronbach’s *α* = 0.83. It showed high correlations with a mapped Strengths and Difficulties Questionnaire hyperactivity subscale (*r* = 0.74, *P* < 0.001) and an ADHD proxy derived from the same cohort (*r* = 0.82, *P* < 0.001), supporting construct validity. The binary variable showed comparable prevalence and composition (in terms of gender and subtype) to meta-analytic estimates of ADHD in the general population, supporting criterion validity.

The dimensional measure was used in the main analyses to capture the full population range of ADHD traits and to improve power. A detailed user guide describing how measures can be derived is available from the Centre for Longitudinal Studies (CLS). Items included are presented in Supplementary Material 1.

#### Psychological distress

Psychological distress was assessed at ages 26, 30, 34, 42 and 46 using the Malaise Inventory Scale^29^. This questionnaire has shown good internal consistency and convergent validity^29^. At some time points, the full 24-item questionnaire was administered, but at other time points, the 9-item short form was used. To maintain consistency in assessment over time, the nine items from the short form were used for all time points. At each time point, a sum score indicating the number of items cohort members endorsed was derived. In addition, an ‘accumulation’ score was also created. To do this, binary variables were derived for each time point, indicating whether cohort members met or exceeded the threshold for clinically relevant distress (≥4). A cumulative distress measure was created by calculating the proportion of time points at which individuals reported high distress, based on their available data. A proportion score was used instead of a sum score (that is, number of time points with clinically relevant distress) to maximize sample retention among cohort members with missing data. Cohort members with data available for fewer than three time points were excluded from this calculation.

#### Societal exclusion factors

Societal exclusion has previously been conceptualized as a multidimensional construct^30,31^ encompassing key domains captured within BCS70 at age 34 (ref. ^32^): health, relational, political, economic and service exclusion. Measures based on this framework has been validated in BCS70 using data and items from the age 34 sweep^32^, with confirmatory factor analysis supporting its construct validity across domains.

Health exclusion refers to poor health and comprised 4 items: self-assessed health (5-point scale, ranging from excellent to very poor), whether health limits daily activities (3-point scale, including yes, no but health problems, and no and no health problems), life satisfaction (measures on a 0–10 scale) and self-efficacy (a combined score including three items: whether the cohort member gets what they want out of life, has control over their life and can run life as they want). The item indicating risk of depression used in ref. ^32^ was removed owing to overlap between this measure and the study outcome.

Relational exclusion refers to lack of emotional support and distrust in others. This was made up of 3 items: relationship status (currently in a relationship, or not), trust in people (4-point scale, ranging from ‘a lot’ to ‘not at all’) and emotional support (number of people cohort member reported being able to turn to for support).

Political exclusion refers to minimal or no political engagement, and comprised 4 items: whether cohort member voted in the last general election (yes or no), whether interested in politics (4-point scale, ranging from ‘very interested’ to ‘not at all interested’), belief in influence on decisions affecting local area (4-point scale, ranging from ‘definitely agree’ to ‘definitely disagree’) and active participation (self-report of contact with governments, attending public meetings, participation in public demonstration or signing petitions in the last year).

For parsimony and owing to high correlations between income and employment, resource and labor market exclusion were combined into one domain indicating economic exclusion. Economic exclusion refers to financial instability and unemployment, and was comprised of 5 items: income poverty (reported income below 60% of median income), subjective poverty (5-point scale, ranging from ‘living comfortably’ to ‘finding it very difficult’), saving opportunities (self-report of whether cohort member saves any amount of their income), credit market exclusion (self-report of whether cohort member borrowed money from pawnbroker, money lender, friends or family in the last year) and economic activity (employed or unemployed).

Services exclusion refers to limited access to high-quality public services. This was based on cohort members’ ratings of public services in their area, including social and leisure facilities, health services, education services, police services and public transport services. These items were all scored on a five-point scale, ranging from ‘very good’ to ‘very poor’.

In line with the validated model in previous research^32^, each item was dichotomized to indicate high (1) versus low (0) exclusion. Thresholds used for dichotomization are available elsewhere^32^. Next, a summary score was created for each domain of societal exclusion by summing the relevant indicators, with higher scores reflecting greater levels of exclusion.

#### Covariates

Covariates included sex assigned at birth, ethnicity and social class at age 10. Sex was classified as male or female. Ethnicity was categorized as white or minoritized ethnicity. Owing to the small sample size of minoritized ethnic groups in this sample (<3%), a more detailed breakdown was not possible. Social class at age 10 was determined based on the father’s occupation, or the mother’s occupation if this was unavailable. Classification followed the Registrar General’s Social Class (RGSC) framework, which includes unskilled, partly skilled, manual, non-manual, managerial and technical, and professional categories^33^.

### Statistical analyses

The sample with missing data was compared with the complete-case sample to assess differences in key variables and covariates, using *t*-tests and *χ*^2^ tests as appropriate.

First, linear regression models were used to test whether ADHD traits were associated with cumulative distress from ages 26 to 46 (proportion of time points in which clinically relevant distress was reported). Next, growth curve models were fit to the data to model distress scores from ages 26 to 46. Linear and quadratic models were fitted and compared based on standard fit indices (CFI and TLI ≥ 0.95, RMSEA ≤ 0.06, lower *χ*^2^ values indicating better fit^25^), with the best fitting model used in subsequent analyses. Next, growth mixture models were fitted to identify distinct trajectories of distress. Models with a 2-, 3-, 4-, 5- and 6-class solution were fitted and compared using a range of standard indices, including AIC and BIC (lower scores indicating better fit), entropy (values closer to 1 indicating better classification, and a threshold of ≥0.80 used to indicate acceptable entropy), percentages in each class (to avoid small and unstable groups) and Lo–Mendell–Rubin adjusted likelihood ratio test (to test whether there is significant improvement in fit compared with a model with one less class). Once the optimal number of trajectories was identified, class membership was extracted to use in subsequent analyses. Multinomial logistic regressions were run to test associations between childhood ADHD traits and distress trajectory class membership. The class with the largest sample size was used as the reference category.

Predicted probabilities of having clinically relevant distress at age 46 were estimated using the margins command following adjusted logistic regression in Stata, comparing individuals with and without high ADHD traits, using the binary indicator.

Finally, path models were run to test (1) direct effects of ADHD traits on distress at age 46, and (2) indirect effects through domains of adult societal exclusion: health, relational, political, economic and services. Covariances were included between societal exclusion domains to account for known correlations. Model fit was assessed using standard fit indices (*χ*^2^, CFI, TLI and RMSEA)^34^.

Models were run unadjusted (model 1) and adjusted for key covariates (sex, ethnicity and social class at age 10) (model 2). Missing data were dealt with in trajectory models and path models using full information maximum likelihood. Analyses were conducted in Stata v18 and MPlus v8.

### Reporting summary

Further information on research design is available in the Nature Portfolio Reporting Summary linked to this article.

## Supplementary information

Supplementary Information Supplementary Materials 1 and 2, Tables 1–5 and Fig. 1.

Reporting Summary

## Data Availability

Data are available from the UK Data Service at https://ukdataservice.ac.uk/.
