# Supplementary material for: Childhood attention deficit hyperactivity disorder traits, societal exclusion and midlife psychological distress
Source: Nat Ment Health. 2026 Mar 27;4(4):566–73. doi: 10.1038/s44220-026-00600-0 (PMC13076204; doi:10.1038/s44220-026-00600-0)

nature mental health

Supplementary information https://doi.org/10.1038/s44220-026-00600-0

Childhood attention deficit hyperactivity disorder traits, societal exclusion and midlife psychological distress

In the format provided by the authors and unedited

Supplementary Materials

Supplementary Materials 1: Items included in measures of ADHD traits.

Supplementary Materials 2: Detailed information about growth mixture model comparisons.

Supplementary Table 1: Missing data analysis.

Supplementary Table 2: Correlation matrix for key variables included in analyses.

Supplementary Table 3: Growth mixture model class comparisons.

Supplementary Table 4: Associations between ADHD traits at age 10 and proportion of time-points with clinically relevant psychological distress up to age 46 (linear regression), including results for covariates.

Supplementary Table 5: Associations between ADHD traits at age 10 and psychological distress class membership, including results for covariates.

Supplementary Figure 1: Unadjusted path model testing the role of societal exclusion in the association between ADHD traits and midlife psychological distress.

Supplementary Materials 1: Items included in measures of ADHD traits.

DSM-5 criteria	Corresponding items in BCS70
**Inattentive**	
Often fails to give close attention to details or makes careless mistakes in schoolwork, at work, or with other activities.	N/A
Often has trouble holding attention on tasks or play activities.	R-j155 - Pays attention to what is being explained in class<br>m82 - Has difficulty concentrating on any particular task though may return to it frequently<br>j129 - Cannot concentrate on any particular task, even though the child may return to it frequently<br>j077 - How well does this child concentrate on educational tasks, in comparison with the average 10-year-old?
Often does not seem to listen when spoken to directly.	N/A
Often does not follow through on instructions and fails to finish schoolwork, chores, or duties in the workplace (e.g., loses focus, side-tracked).	m76 - Fails to finish things he/she starts, short attention span<br>R-j174 - Child completes tasks which are started<br>j177 - Fails to finish things he starts
Often has trouble organizing tasks and activities.	N/A
Often avoids, dislikes, or is reluctant to do tasks that require mental effort over a long period of time (such as schoolwork or homework).	R-j139 - Shows perseverance; persists with difficult or routine work
Often loses things necessary for tasks and activities (e.g. school materials, pencils, books, tools, wallets, keys, paperwork, eyeglasses, mobile telephones).	N/A
Is often easily distracted	m65 – Inattentive, easily distracted<br>j152 – Is easily distracted
Is often forgetful in daily activities.	j158 – Is forgetful when given a complex task
**Hyperactive**	

Often fidgets with or taps hands or feet, or squirms in seat.	m44 - Is squirmy or fidgety<br>j151 - Squirmy and fidgety<br>m77 - Given to rhythmic tapping or kicking<br>j165 - Given to rhythmic tapping or rhythmic kicking during class<br>j082 - What percentage of the time is the child fidgeting and indulging other minor distracting activities, when he/she is expected to be working?
Often leaves seat in situations when remaining seated is expected.	j081 - What percentage of the time is the child moving around the classroom, when he/she is expected to be working? (paraphrased)
Often runs about or climbs in situations where it is not appropriate (adolescents or adults may be limited to feeling restless).	m43 - Very restless. Often running or jumping up and down. Hardly ever still.
Often unable to play or take part in leisure activities quietly.	m57 - Cannot settle to do anything for more than a few moments
Is often "on the go" acting as if "driven by a motor".	m72 – Shows restless or overactive behaviour<br>j150 - Shows restless or overactive behaviour
Often talks excessively.	j080 - What percentage of the time is the child talking to other children, when he/she is expected to be working?
Often blurts out an answer before a question has been completed.	m73 – Is impulsive, excitable
Often has trouble waiting his/her turn.	m71 - Requests must be met immediately, easily frustrated<br>j175 - Requests must be met immediately - easily frustrated
Often interrupts or intrudes on others (e.g., butts into conversations or games).	m74 - Interferes with the activity of other children<br>j142 - Interferes with the activities of other children
**Conditional criteria**	
Several inattentive or hyperactive-impulsive symptoms were present before age 12 years.	All criteria evaluated at age 10
Several symptoms are present in two or more settings, (such as at home, school or work; with friends or relatives; in other activities).	If both mother and teacher indicated 4+ symptoms were observed often.

There is clear evidence that the symptoms interfere with, or reduce the quality of, social, school, or work functioning.	N/A
The symptoms are not better explained by another mental disorder (such as a mood disorder, anxiety disorder, dissociative disorder, or a personality disorder).	Excluded if diagnosed with another psychiatric disorder, identified using ICD codes in the medical questionnaire: 295, 296, 300, 301.
The symptoms do not happen only during the course of schizophrenia or another psychotic disorder.	Excluded if diagnosed with another psychiatric disorder, identified using ICD codes in the medical questionnaire: 295, 296, 300, 301.

Supplementary Materials 2: Details information about growth mixture model comparisons.

Growth mixture models were fitted to the data to compare the 2-, 3-, 4-, 5-, and 6-class models. Results showed that the 4-class model outperformed the 3-class model, with notably lower AIC and BIC scores (AIC=167271.69, BIC=167399.35), a significant Lo-Mendell-Rubin adjusted LRT test ($p<.0001$), and acceptable entropy levels (0.80). The Lo-Mendell-Rubin adjusted LRT test was attenuated ($p=.001$) for the 5-class model and non-significant ($p=0.33$) for the 6-class model. In addition, entropy is lower than the recommended threshold for the 5-class (0.78) and 6-class (0.77) models. Smaller sample sizes in some of the classes included in the 5-class and 6-class models may raise concerns about stability and interpretability. As such, the 4-class model was selected as the optimal solution as it fit significantly better than the 3-class model, maintained reasonable sample sizes and entropy, and avoided issues with instability and interpretability which may arise in models with more classes. In addition, the trajectories identified in the 4-class model were theoretically meaningful, and in line with previous research deriving similar trajectories in these data.

Supplementary Table 1: Missing data analysis.

		Excluded due to missing data	Included in main model	Difference
ADHD traits, Mean (SD)		0.07 (0.92)	-0.12 (0.85)	t(14745)=12.68, *p*<.001
Psychological distress at age 46		1.80 (2.15)	1.75 (2.11)	t(7776)=0.79, *p*=.43
Health exclusion, Mean (SD)		0.63 (0.93)	0.56 (0.90)	t(9564)=2.72, *p*=.007
Relational exclusion, Mean (SD)		0.57 (0.69)	0.53 (0.67)	t(9581)=2.21, *p*=.03
Political exclusion, Mean (SD)		2.20 (1.13)	2.17 (1.15)	t(9562)=0.93, *p*=.35
Economic exclusion, Mean (SD)		1.21 (1.32)	1.06 (1.22)	t(8356)=4.04, *p*<.001
Service exclusion, Mean (SD)		0.76 (1.03)	0.71 (1.01)	t(7141)=1.68, *p*=.09
Sex, N (%)				
	Male	3,206 (57.37)	4,502 (48.51)	$\chi^2(1)$=109.67, *p*<.001
	Female	2,382 (42.63)	4,778 (51.49)	
Ethnicity, N (%)				
	White	4,165 (94.02)	9,007 (97.06)	$\chi^2(1)$=73.51, *p*<.001
	Minority ethnicity	265 (5.98)	273 (2.94)	
Social class at age 10				
	Unskilled	241 (6.10)	350 (3.77)	$\chi^2(5)$=132.28, *p*<.001
	Partly skilled	634 (16.06)	1,202 (12.95)	
	Manual	1,762 (44.63)	3,752 (40.43)	
	Non-manual	376 (9.52)	1,040 (11.21)	
	Managerial and technical	755 (19.12)	2,343 (25.25)	
	Professional	180 (4.56)	593 (6.39)	

Supplementary Table 2: Correlation matrix for key variables included in analyses.

	ADHD traits age 10	Distress age 26	Distress age 30	Distress age 34	Distress age 42	Distress age 46	Health exclusion age 34	Relational exclusion age 34	Political exclusion age 34	Economic exclusion age 34	Service exclusion age 34
ADHD traits age 10	1										
Distress age 26	0.10 (<.001)*	1									
Distress age 30	0.09 (<.001)	0.57 (<.001)	1								
Distress age 34	0.10 (<.001)	0.51 (<.001)	0.59 (<.001)	1							
Distress age 42	0.09 (<.001)	0.45 (<.001)	0.49 (<.001)	0.57 (<.001)	1						
Distress age 46	0.09 (<.001)	0.45 (<.001)	0.47 (<.001)	0.54 (<.001)	0.64 (<.001)	1					
Health exclusion age 34	0.14 (<.001)	0.29 (<.001)	0.36 (<.001)	0.51 (<.001)	0.35 (<.001)	0.35 (<.001)	1				
Relational exclusion age 34	0.10 (<.001)	0.14 (<.001)	0.16 (<.001)	0.20 (<.001)	0.13 (<.001)	0.14 (<.001)	0.31 (<.001)	1			
Political exclusion age 34	0.09 (<.001)	0.03 (.01)	0.03 (.001)	0.05 (<.001)	0.06 (<.001)	0.04 (<.001)	0.07 (<.001)	0.10 (<.001)	1		
Economic exclusion age 34	0.11 (<.001)	0.22 (<.001)	0.23 (<.001)	0.28 (<.001)	0.21 (<.001)	0.22 (<.001)	0.34 (<.001)	0.16 (<.001)	0.09 (<.001)	1	
Service exclusion age 34	0.07 (<.001)	0.08 (<.001)	0.12 (<.001)	0.16 (<.001)	0.12 (<.001)	0.12 (<.001)	0.16 (<.001)	0.17 (<.001)	0.03 (.01)	0.07 (<.001)	1

Presented as correlation coefficient (p).

Supplementary Table 3: Growth mixture model class comparisons.

	**2 class**	**3 class**	**4 class**	**5 class**	**6 class**
	11	14	**17**	20	23
AIC	170339.10	168340.67	**167271.69**	166742.16	166387.41
BIC	170421.71	168445.80	**167399.35**	166892.35	166560.13
Sample size adjusted BIC	170386.75	168401.31	**167345.33**	166828.79	166487.04
Entropy	0.82	0.82	**0.80**	0.78	0.77
Smallest % in a class	15.30	8.02	**3.84**	3.17	2.17
Lo-Mendell-Rubin adjusted LRT test	3129.40	1936.55	**1038.58**	517.39	348.53
Lo-Mendell-Rubin adjusted LRT test p value	<.0001	<.0001	**<.0001**	0.0007	0.3294

Supplementary Table 4: Associations between ADHD traits at age 10 and proportion of time-points with clinically relevant psychological distress up to age 46 (linear regression), including results for covariates.

	**Model 1: Unadjusted**	**Model 2: Fully adjusted**
ADHD traits at age 10	0.11 (0.09-0.13), <.001*	0.13 (0.10-0.15), <.001
Sex		
Male	-	*REF*
Female	-	0.30 (0.26-0.35), <.001
Ethnicity		
White	-	*REF*
Minoritised ethnicity	-	0.04 (-0.10-0.18), .56
Social class at age 10		
Unskilled	-	*REF*
Partly skilled	-	-0.13 (-0.26-0.001), .05
Manual	-	-0.17 (-0.29- -0.05), .006
Non manual	-	-0.21 (-0.34- -0.08), .002
Managerial and technical	-	-0.23 (-0.36- -0.11), <.001
Professional	-	-0.28 (-0.42- -0.14), <.001

Supplementary Table 5: Associations between ADHD traits at age 10 and psychological distress class membership, including results for covariates.

	**Model 1: Unadjusted**	**Model 2: Fully adjusted**
**Class 2: Moderate and decreasing distress***		
ADHD traits at age 10	1.25 (1.17-1.34), <.001**	1.38 (1.28-1.48), <.001
Sex		
Male	-	*REF*
Female	-	2.41 (2.11-2.75), <.001
Ethnicity		
White	-	*REF*
Minoritised ethnicity	-	1.65 (1.23-2.21), .001
Social class at age 10		
Unskilled	-	*REF*
Partly skilled	-	0.86 (0.62-1.20), .37
Manual	-	0.84 (0.62-1.14), .26
Non manual	-	0.84 (0.60-1.19), .33
Managerial and technical	-	0.77 (0.56-1.06), .11
Professional	-	0.78 (0.53-1.15), .20
**Class 3: Low and increasing distress**		
ADHD traits at age 10	1.23 (1.15-1.31), <.001	1.30 (1.21-1.40), <.001
Sex		
Male	-	*REF*
Female	-	1.75 (1.55-1.99), <.001
Ethnicity		
White	-	*REF*
Minoritised ethnicity	-	0.84 (0.58-1.22), .37
Social class at age 10		

Unskilled	-	*REF*
Partly skilled	-	0.94 (0.68-1.31), .73
Manual	-	0.86 (0.63-1.17), .33
Non manual	-	0.83 (0.58-1.17), .28
Managerial and technical	-	0.85 (0.62-1.16), .31
Professional	-	0.65 (0.44-1.97), .04
**Class 4: Persistently high distress**		
ADHD traits at age 10	1.46 (1.32-1.62), <.001	1.46 (1.31-1.64), <.001
Sex		
Male	-	*REF*
Female	-	1.63 (1.33-2.00), <.001
Ethnicity		
White	-	*REF*
Minoritised ethnicity	-	0.84 (0.46-1.51), .55
Social class at age 10		
Unskilled	-	*REF*
Partly skilled	-	0.71 (0.46-1.11), .14
Manual	-	0.55 (0.37-0.83), .004
Non manual	-	0.51 (0.31-0.83), .007
Managerial and technical	-	0.43 (0.28-0.68), <.001
Professional	-	0.23 (0.11-0.47), <.001

Supplementary Figure 1: Unadjusted path model testing the role of societal exclusion in the association between ADHD traits and midlife psychological distress.

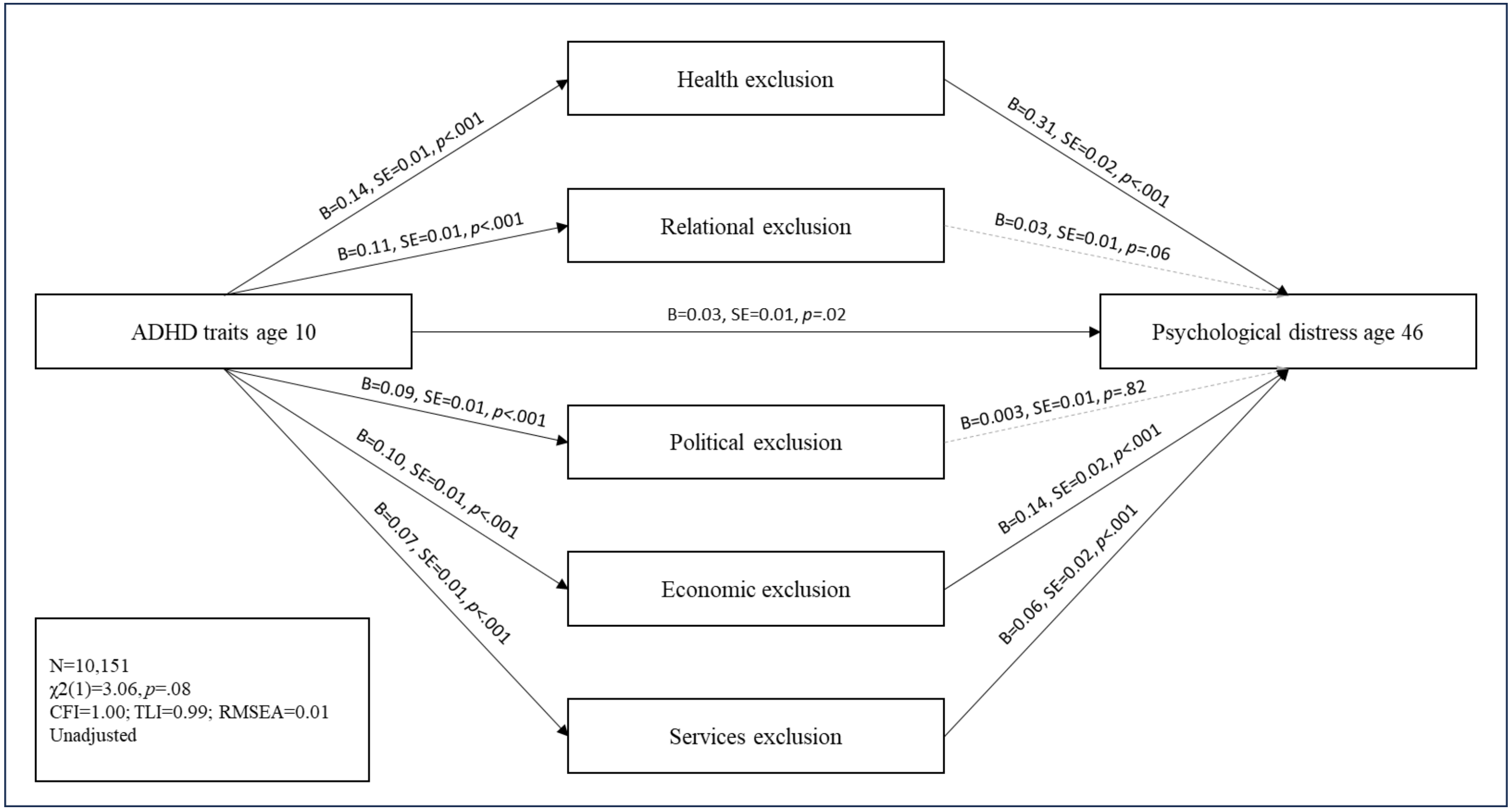

Supplement: Supplementary file 1 — Supplementary Materials 1 and 2, Tables 1–5 and Fig. 1. [file 44220_2026_600_MOESM1_ESM.pdf]
