# Supplementary material for: Childhood attention deficit hyperactivity disorder traits, societal exclusion and midlife psychological distress
Source: Nat Ment Health. 2026 Mar 27;4(4):566–73. doi: 10.1038/s44220-026-00600-0 (PMC13076204; doi:10.1038/s44220-026-00600-0)
Supplement: Supplementary file 2 — Reporting Summary [file 44220_2026_600_MOESM2_ESM.pdf]

nature portfolio

Corresponding author(s): Amber John

Last updated by author(s): 22/10/2025

Reporting Summary

Nature Portfolio wishes to improve the reproducibility of the work that we publish. This form provides structure for consistency and transparency in reporting. For further information on Nature Portfolio policies, see our Editorial Policies and the Editorial Policy Checklist.

Statistics

For all statistical analyses, confirm that the following items are present in the figure legend, table legend, main text, or Methods section.

n/a	Confirmed	
☐	☒	The exact sample size (*n*) for each experimental group/condition, given as a discrete number and unit of measurement
☐	☒	A statement on whether measurements were taken from distinct samples or whether the same sample was measured repeatedly
☐	☒	The statistical test(s) used AND whether they are one- or two-sided<br>*Only common tests should be described solely by name; describe more complex techniques in the Methods section.*
☐	☒	A description of all covariates tested
☐	☒	A description of any assumptions or corrections, such as tests of normality and adjustment for multiple comparisons
☐	☒	A full description of the statistical parameters including central tendency (e.g. means) or other basic estimates (e.g. regression coefficient) AND variation (e.g. standard deviation) or associated estimates of uncertainty (e.g. confidence intervals)
☐	☒	For null hypothesis testing, the test statistic (e.g. F, t, r) with confidence intervals, effect sizes, degrees of freedom and P value noted<br>*Give P values as exact values whenever suitable.*
☒	☐	For Bayesian analysis, information on the choice of priors and Markov chain Monte Carlo settings
☒	☐	For hierarchical and complex designs, identification of the appropriate level for tests and full reporting of outcomes
☐	☒	Estimates of effect sizes (e.g. Cohen's *d*, Pearson's *r*), indicating how they were calculated

Our web collection on statistics for biologists contains articles on many of the points above.

Software and code

Policy information about availability of computer code

Data collection	N/A. No data collection.
Data analysis	Stata v18, MPlus v8

For manuscripts utilizing custom algorithms or software that are central to the research but not yet described in published literature, software must be made available to editors and reviewers. We strongly encourage code deposition in a community repository (e.g. GitHub). See the Nature Portfolio guidelines for submitting code & software for further information.

Data

Policy information about availability of data

All manuscripts must include a data availability statement. This statement should provide the following information, where applicable:

- Accession codes, unique identifiers, or web links for publicly available datasets
- A description of any restrictions on data availability
- For clinical datasets or third party data, please ensure that the statement adheres to our policy

Data are available from the UK Data Service.

Research involving human participants, their data, or biological material

Policy information about studies with human participants or human data. See also policy information about sex, gender (identity/presentation), and sexual orientation and race, ethnicity and racism.

Reporting on sex and gender	Information on sex at birth are available in these data, and are correctly referred to as 'sex' throughout the manuscript.
Reporting on race, ethnicity, or other socially relevant groupings	Ethnicity was categorised as white or minoritised ethnicity. Due to the small sample size of minoritised ethnic groups in this sample (<3%), a more detailed breakdown was not possible. This is described in the manuscript.
Population characteristics	A total of 9,280 people were included in the main path model. All participants were born in 1970 and were 46 at the latest data sweep. The sample are 48.51% men and 51.49% women.
Recruitment	Participants were recruited as all individuals born in England, Scotland, and Wales during a single week in 1970, forming a nationally representative birth cohort study.
Ethics oversight	This secondary data study is covered under the original cohort ethical approval. Ethical approval for the most recent wave of data collection was obtained from the South East Coast - Brighton and Sussex MREC (15/LO/1446).

Note that full information on the approval of the study protocol must also be provided in the manuscript.

Field-specific reporting

Please select the one below that is the best fit for your research. If you are not sure, read the appropriate sections before making your selection.

☐ Life sciences ☒ Behavioural & social sciences ☐ Ecological, evolutionary & environmental sciences

For a reference copy of the document with all sections, see nature.com/documents/nr-reporting-summary-flat.pdf

Behavioural & social sciences study design

All studies must disclose on these points even when the disclosure is negative.

Study description	This is a quantitative secondary data analysis of the 1970 British Cohort Study (BCS70). BCS70 is a population-based, nationally-representative longitudinal cohort study of 17,198 individuals born in England, Scotland, and Wales during a single week in 1970
Research sample	A total of 9,280 people were included in the main path model. All participants were born in 1970 and were 46 at the latest data sweep. The sample are 48.51% men and 51.49% women. The sample was nationally representative at birth
Sampling strategy	BCS70 used a population-based sampling strategy, including all live births in England, Scotland, and Wales during a single week in 1970 to create a nationally representative cohort.
Data collection	Data have been collected through multiple follow-up sweeps from birth to age 46, using a combination of interviews, self-completion questionnaires, cognitive assessments, and biomedical measurements.
Timing	Data collection began in 1970 and follow up data have been collected over 46 years.
Data exclusions	No data exclusions were made.
Non-participation	The original cohort comprised 17,198 people at birth. The main analytic sample for this study included 9,280 people with data available.
Randomization	N/A

Reporting for specific materials, systems and methods

We require information from authors about some types of materials, experimental systems and methods used in many studies. Here, indicate whether each material, system or method listed is relevant to your study. If you are not sure if a list item applies to your research, read the appropriate section before selecting a response.

Materials & experimental systems

n/a	Involved in the study
☒	☐ Antibodies
☒	☐ Eukaryotic cell lines
☒	☐ Palaeontology and archaeology
☒	☐ Animals and other organisms
☒	☐ Clinical data
☒	☐ Dual use research of concern
☒	☐ Plants

Methods

n/a	Involved in the study
☒	☐ ChIP-seq
☒	☐ Flow cytometry
☒	☐ MRI-based neuroimaging

Plants

Seed stocks	N/A
Novel plant genotypes	N/A
Authentication	N/A
